# Recent Advances in the Study of Na^+^/K^+^-ATPase in Neurodegenerative Diseases

**DOI:** 10.3390/cells11244075

**Published:** 2022-12-16

**Authors:** Xiaoyan Zhang, Weithye Lee, Jin-Song Bian

**Affiliations:** 1 Department of Pharmacology, School of Medicine, Southern University of Science and Technology, Shenzhen 518055, China; 2 Department of Pharmacology, Yong Loo Lin School of Medicine, National University of Singapore, Singapore 117600, Singapore

**Keywords:** Na^+^/K^+^-ATPase, neurodegenerative diseases, protein–protein interaction, mitochondrial dysfunction, oxidative stress

## Abstract

Na^+^/K^+^-ATPase (NKA), a large transmembrane protein, is expressed in the plasma membrane of most eukaryotic cells. It maintains resting membrane potential, cell volume and secondary transcellular transport of other ions and neurotransmitters. NKA consumes about half of the ATP molecules in the brain, which makes NKA highly sensitive to energy deficiency. Neurodegenerative diseases (NDDs) are a group of diseases characterized by chronic, progressive and irreversible neuronal loss in specific brain areas. The pathogenesis of NDDs is sophisticated, involving protein misfolding and aggregation, mitochondrial dysfunction and oxidative stress. The protective effect of NKA against NDDs has been emerging gradually in the past few decades. Hence, understanding the role of NKA in NDDs is critical for elucidating the underlying pathophysiology of NDDs and identifying new therapeutic targets. The present review focuses on the recent progress involving different aspects of NKA in cellular homeostasis to present in-depth understanding of this unique protein. Moreover, the essential roles of NKA in NDDs are discussed to provide a platform and bright future for the improvement of clinical research in NDDs.

## 1. Introduction

Neurodegenerative diseases (NDDs) are a group of diseases characterized by chronic, progressive and irreversible neuronal loss in specific brain areas [1]. Depletion of neurons in the brain is accompanied by physical, psychosocial, emotional and cognitive dysfunction in the affected individuals [2]. NDDs encompass Alzheimer’s disease (AD), Parkinson’s disease (PD), amyotrophic lateral sclerosis (ALS), Huntington’s disease (HD), multiple sclerosis (MS), etc. [3]. As the average age and life expectancy increase, the incidence of NDDs increases [4]. However, there are no disease-modifying therapies to slow NDD progression.

NKA dysfunction was found in experimental NDDs [5,6]. Restoration of the activity and membrane stability of NKA could relieve neurological disability [7]. Moreover, NKA dysfunction was also related to various pathological properties of NDDs, such as misfolded and aggregated proteins, mitochondrial dysfunction and oxidative stress [8]. In addition, our recent study demonstrated that an NKA-stabilizing monoclonal antibody, DR5-12D, could stabilize membrane NKA and protect the brain against α-syn-induced pathology [9]. In short, NKA might serve as a promising therapeutic modality for NDDs.

As aforementioned, NKA is involved in the development and progression of NDDs. In this review, the current progress regarding the roles of NKA will be discussed. In addition, the pathophysiological properties of NDDs and the barriers of NKA in these diseases will also be clarified.

## 2. Na^+^/K^+^-ATPase

### 2.1. The Discovery of NKA

Action potential is an electrical phenomenon in the excitable cell membrane, which propagates signals without attenuation [10]. Sir Alan Lloyd Hodgkin and Sir Andrew Fielding Huxley discovered its mechanism and then shared the 1963 Nobel Prize [11]. Scientists hypothesized that mammalian cells require a “sodium pump” to produce an ionic gradient for an action potential [12]. Over a century after the discovery of the action potential, the Danish biochemist Jens Christian Skou identified a novel ATPase that relied on the presence of Na^+^ and K^+^. Furthermore, this ATPase could be specifically inhibited by ouabain (an inhibitor of sodium pumps), demonstrating that it was the “sodium pump” [13]. Hence, Skou and two other scientists shared the 1997 Nobel Prize in chemistry for their work on ATPase [14].

### 2.2. The Structure, Subunits and Distribution of NKA

**Figure 1:**
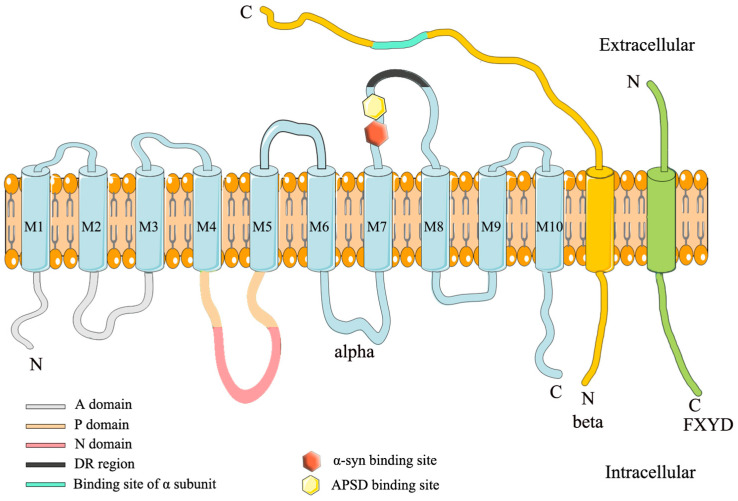
Schematic illustration of NKA structure. NKA contains the α, β and γ subunits. The extracellular region^897^ DVEDSYGQQWTYEQR^911^ (DR region) of the α subunit is associated with the β subunit. The hexagons (yellow and red) depicted above are two binding sites for α-synuclein fibrils and spherical amyloid β (Aβ) oligomers that are close to the DR region [25,26].

NKA belongs to the P-type ATPase family with an acyl-phosphate intermediate. NKA contains three different subunits, α, β and γ (FXYD), with equimolar stoichiometry [15]. Figure 1 illustrates the structure of NKA. The α subunit has 10 transmembrane regions and 3 cytosolic domains in the N-terminus: N-domain (nucleotide binding), P-domain (phosphorylation) and A-domain (actuator) [16]. Since it contains an ATP binding site, the α subunit is also the catalytic subunit [16,17]. There are four isoforms of the α subunit: α1-α4. The α1 is ubiquitously expressed in all mammalian tissues, and its mutation is fatal; the α2 is mainly expressed in glial cells in the brain, and its mutations cause various diseases such as familial hemiplegic migraine type 2 (FHM2), alternating hemiplegia of childhood 1 (AHC1) and epilepsy [18,19]; the α3 is neuron-specific in the brain, and its mutations cause rapid-onset dystonia-parkinsonism (RDP), cerebellar ataxia, sensorineural hearing loss (CAPOS), etc. [16,20,21,22,23]; the α4 is only expressed in the testis, and its stability is vital for male fecundity [24].

The β subunit is a single transmembrane glycoprotein that is smaller than the α subunit [27]. Although it does not form the transport pore of NKA, the β subunit influences the NKA kinetics. As a molecular chaperone, it assists in the correct assembly and membrane delivery of NKA and maintains cell adhesion and cell polarity [28,29,30,31]. The β subunit has four isoforms: β1–β4. The β1 is extensively expressed, whereas β2 is expressed in glial cells, microvessels and the choroid plexus in the brain. The β3 is expressed in microsomes in the brain [27].

The γ subunit, also called FXYD protein, is not required for enzymatic activity, but it modulates NKA catalytic properties [32,33]. In mammals, the FXYD subunit comprises seven isoforms, among them, FXYD2 is the first subunit that regulates NKA [34]. Multiple isoform combinations of α, β and γ subunits show distinctive NKA activity, which could satisfy the needs of distinct tissues and cells [12].

### 2.3. The Function of NKA

As a pump protein, NKA modulates the ionic gradient of Na^+^ and K^+^ to determine resting membrane potential [35]. In addition, the ionic gradient affects osmotic pressure, cell volume and provides energy for the secondary transport of neurotransmitters and other ions [36,37]. Furthermore, NKA also forms a macromolecular complex with other proteins to trigger many signaling pathways [38,39]. For example, NKA binds to different amyloid β peptides to induce neuronal loss and vascular dysfunctions in AD rodent models [40].

## 3. The Role of NKA in Alzheimer’s Disease

Alzheimer’s disease (AD) is an irreversible serious neurodegenerative disorder that was first described by Alois Alzheimer in 1907 [41]. AD patients suffer from cognitive deficit, memory dysfunction and emotional impairment [42]. AD is characterized by the accumulation of extracellular amyloid β (Aβ) plaques and intracellular tau neurofibrillary tangles in the brain [43]. Risk factors for AD include age, family history, gender, education level and comorbidities [44,45,46]. Cumulative evidence suggested that NKA activity was reduced in the brain of AD patients and AD rodent models, which suggests that NKA may be associated with the development of AD [47,48].

### 3.1. NKA and Aβ Plaques

The presence of Aβ plaques is a vital characteristic of AD. Aβ plaques are mainly composed of Aβ peptides [49]. They are manifested by the inequivalent balance between production, clearance and aggregation of peptides, which causes Aβ peptides to accumulate [50]. Aβ precursor protein (APP) is cleaved by β-secretase and γ-secretase to produce Aβ peptides [51]. APP may also be cleaved by α-secretase and γ-secretase to produce soluble amyloid precursor protein α (sAPPα) [52]. It is interesting to observe that NKA acted as a modulator for both Aβ peptides and sAPPα to modulate learning and memory in AD [52]. It was implied in recent studies that sAPPα recruited clusters of NKAα3 at neuronal surfaces. The interaction of sAPPα and NKAα3 regulated the level of intracellular Na^+^ and Ca^2+^ that is required for APP to reach the cell surfaces. When APP interacted with sAPPα in the membrane, the APP/sAPPα complex triggered a cascade of events promoting sAPPα-induced axonal outgrowth [52]. However, the expression levels of sAPPα and NKA were significantly decreased in an AD rodent model compared with the control group [53]. The sAPPα deficiency reduced the neuroprotective effect of sAPPα. In contrast, the activity and expression level of β-secretase were increased in AD patients and rodent models, which produced more Aβ peptides [54]. The overproduction of Aβ peptides influences the activity and expression level of NKA through downstream pathways.

#### 3.1.1. Interactions between NKA and Aβ Peptides

**Figure 2:**
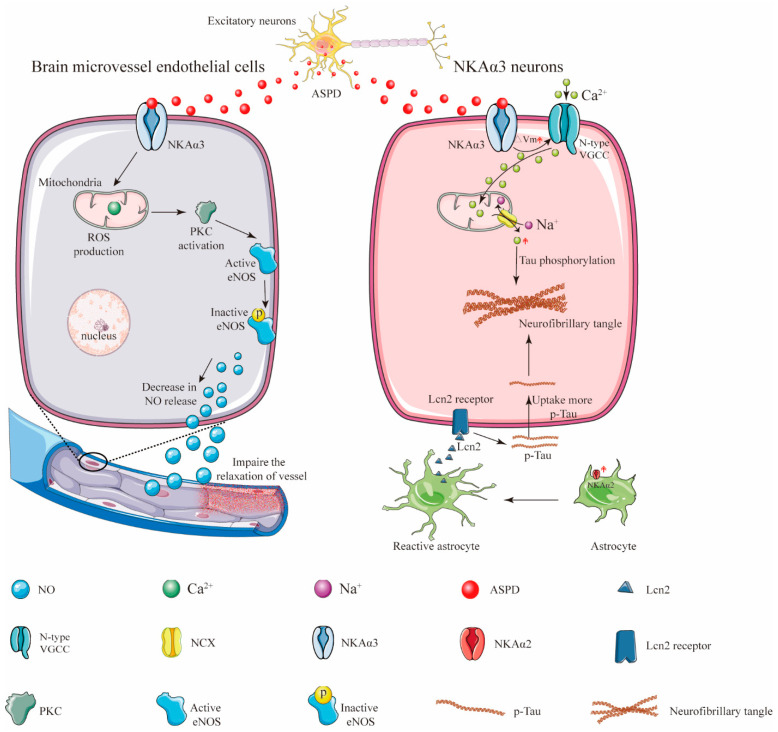
Schematic illustration showing how ASPD secretion from the excitatory neurons induces neuronal loss and vascular pathologies. In response to central nervous system (CNS) damage, astrocytic NKAα2 level was upregulated and contributed to astrogliosis [59]. The activated astrocytes then secreted more proinflammatory protein lipocalin-2 (Lcn-2) to neurons, causing neurons to uptake more extracellular tau and resulting in the formation of the neurofibrillary tangles [59].

Aβ peptides form structurally distinct complexes to exert different toxic functions via different targets [55]. Specifically, amylospheroids (ASPDs) are a population of neurotoxic Aβ aggregates containing approximately 30 Aβ monomers [56]. In a normal physiological condition, full-length APP is either cleaved by β- and γ-secretase cleavages to produce Aβ aggregates or re-internalized via an endosomal/lysosomal degradation pathway [57]. Evidence showed that pharmacological inhibition of proteasome-associated degradation of APP dramatically upregulated intra-neuronal ASPD levels [57] (Figure 2). Then, the ASPD-producing neurons died non-apoptotically. More importantly, ASPDs were secreted and caused the degeneration of adjacent NKAα3-expressing neurons [57]. Compelling studies indicated that NKAα3 was the neuronal death-inducing target of ASPD. The region in the fourth extracellular loop (Ex4) of NKAα3 encompasses residues Asn^879^ and Trp^880^. These residues were found to be essential for ASPD/NKAα3 interaction [33]. It is worth mentioning that the ASPD/NKAα3 complex impaired NKAα3-specific activity; activated N-type voltage-gated calcium channels; and caused mitochondrial calcium dyshomeostasis, tau abnormalities and neurodegeneration [33]. In addition to neuropathological features, 60–90% of AD patients exhibit vascular dysfunction, which may precede the onset of AD [58]. In in vitro blood cell cultures and ex vivo blood vessels, ASPDs bound to NKAα3 in endothelial cells, activating protein kinase C (PKC). The ASPD/NKAα3 complex increased the PKC-phosphorylated inactive form of endothelial nitric oxide synthase (eNOS) and decreased nitric oxide (NO) production. NO deficiency suppressed the relaxation of blood microvessels and might cause a reduction in cerebral blood flow and other vascular dysfunctions in mouse models of AD [58]. As stated above, these observations revealed the mechanisms of Aβ-induced NKAα3 impairment in AD.

However, ASPD do not exist at the early stages of AD and cannot damage NKA at these stages of the disease [40]. Intriguingly, some recent studies conducted at the early stages of AD demonstrated that NKAα1 also bound to monomeric Aβ_42_ to form a tight, equimolar complex. The Aβ_42_/NKAα1 complex disrupted NKA function and activated Src-kinase in neuroblastoma cells [40]. These findings support the notion that Aβ_42_ is a putative regulator of NKA. In line with this, some studies revealed that the phosphorylation of Aβ_42_ at Ser8 (pS8-Aβ) might neutralize some pathogenic properties of Aβ, reverse NKA activity by preventing the binding of Aβ_42_ to NKA, and then reduce cerebral plaque deposition [40]. These amazing and thrilling studies unveil a new outlook towards NKA structural derivatives to prevent the “protein–NKA” interaction for the treatment of AD.

#### 3.1.2. NKA and Oxidative Stress

Aβ deposition caused oxidative stress in neuronal cells, which resulted in severe cell damage in AD patients and rodent models [60]. Methionine at residue 35 (Met35) of the Aβ sequence was required for Aβ-induced oxidative damage in AD [61]. Additional studies indicated that trans fatty acids enhance Aβ-induced oxidative stress in PC12 cells [61]. Following studies found that trans fatty acids reacted with Met35 of the Aβ residue. This complex significantly impaired the activity of NKA via enhancing the generation of ROS and nitric oxide and elevating caspase-3, caspase-8 and nitric oxide synthase activities [61]. Similarly, some studies found that the glutathionylation of the NKA α subunit determined enzyme the redox sensitivity. Meanwhile, glutathionylation of the NKA α subunit depended on the redox status of cells during the enzyme biosynthesis [62]. It is interesting to observe that long-term exposure to Aβ changed the thiol redox status of SH-SY5Y cells and inhibited NKA activity by upregulating the glutathionylation of the α subunit of NKA [56]. These studies indicate that NKA deficiency observed in AD may, at least in part, stem from Aβ-induced oxidative stress. Consistently, some antioxidants, such as 17β-estradiol, genistein, basic fibroblast growth factor (bFGF) and organosulfur compounds exerted their neuroprotective effects by restoring NKA activity in AD rodent models [63,64,65,66]. Thus, the drugs targeted at restoring NKA activity may be a potential therapeutic target in oxidative stress-induced AD.

### 3.2. NKA and Tau

As a microtubule-associated protein, tau affects the assembly and stabilization of the microtubular cytoskeleton [67]. The pathological form of tau is the principal component of paired helical filaments (PHFs) which are further associated with neurofibrillary tangles (NFTs). NFTs are the main hallmarks of AD [67]. Numerous studies reported that tau aggregates could be released from one and spread to other neurons, displaying “prion-like” properties [68]. Evidence showed that exogenous fibrillar tau (Fib-tau) formed clusters on the neuronal surfaces. Fib-tau clusters destabilized NKAα3 and formed a complex with NKAα3. This complex reduced neurons’ capacity to control membrane depolarization and exacerbated neuronal loss in AD [69].

Intriguingly, a recent paper published in 2022 demonstrated that the astrocyte-specific isoform of the NKA α subunit, NKAα2, might positively regulate astrocytic-dependent neuroinflammation [59]. Following evidence demonstrated that the expression level of NKAα2 was elevated in human tauopathies and a mouse model of tauopathy. The pharmacological inhibition of NKAα2 robustly suppressed neuroinflammation and reduced brain atrophy. In addition, NKAα2 knockdown in tauopathy mice halted the accumulation of tau pathology [59]. These observations revealed that NKAα2 promoted tauopathy via increased tau uptake in neurons (Figure 2). Taken together, these findings indicate that the roles of different isoforms of NKA in different brain cells may be different and sophisticated. More evidence are still needed to elucidate the relationship between the isoforms of the NKA α subunit and the progression of AD.

## 4. The Role of NKA in Parkinson’s Disease

Parkinson’s disease (PD) is the second most common neurodegenerative disorder after Alzheimer’s disease, with projections of an increase to around 10 million patients globally by 2030 [70]. The loss of dopamine (DA) neurons in the substantia nigra pars compacta (SNpc) and the formation of Lewy bodies (LBs) in neurons are hallmarks of PD [71]. Depletion of DA in the striatum is accompanied by motor deficits (including bradykinesia and resting tremor) and non-motor features (including constipation, hyposmia, depression, cognitive decline and sleep alterations) [72]. There are currently no disease-modifying treatments to slow PD progression. Ironically, NKA dysfunction exists in PD rodent models and PD patients [73,74]. In addition, mutations in the NKAα3 gene *ATP1A3* were associated with rapid-onset dystonia-parkinsonism (RDP) [75]. Consistently, restoration of activity and membrane stability of the NKA in neurons relieved dopaminergic neurodegeneration. These results indicate a strong correlation between NKA and the pathogenesis of PD. Herein, we focus on recent research progress involving different aspects of NKA in the progression of PD.

### 4.1. NKA and α-Synuclein

**Figure 3:**
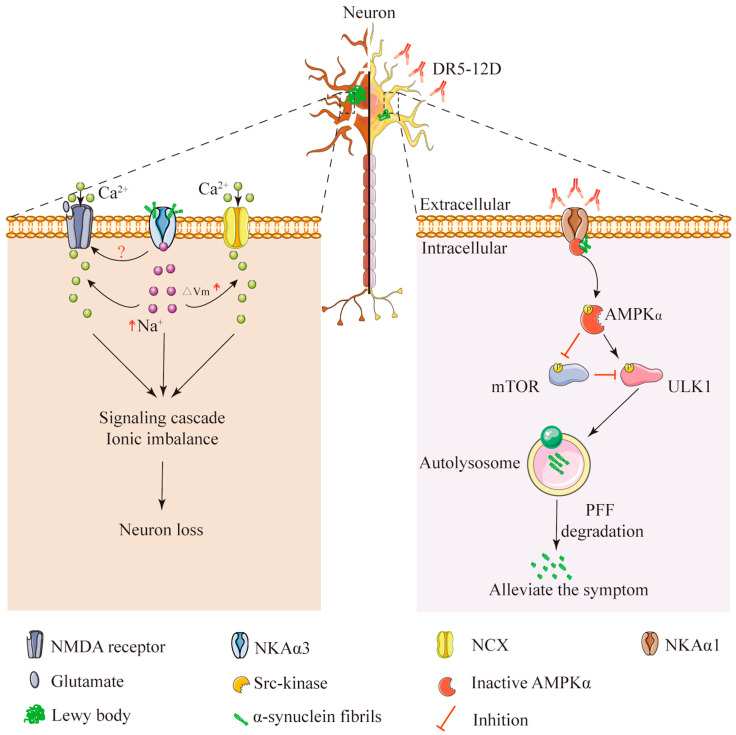
Interactions between α-syn and NKA α subunit in the extracellular and intracellular segments. Left panel: α-syn bound to the neuronal membrane and formed a complex with NKAα3. This complex impaired NKAα3 activity and increased glutamate-induced Ca^2+^ influx to induce neuronal loss. Right panel: Intracellular part of NKAα1 formed a complex with α-syn/AMPKα to translocate AMPKα to the plasma membrane. DR5-12D prevented the formation of this complex and alleviated the symptoms of PD.

Several genes whose mutations cause inherited PD have been widely studied and verified [76]. These include *SNCA*, which encodes a small protein, α-synuclein (α-syn) [77]. In a normal physiological condition, α-syn regulates the release of neurotransmitters [78]. However, some mutations of *SNCA*, such as A53T and A30P, inhibit the degradation of α-syn, leading to α-syn accumulation. When the balance between the production and clearance of α-syn is disrupted, the soluble monomeric α-syn aggregates and misfolds into oligomers and then forms Lewy bodies (LBs) in neurons. The presence of LBs is the major pathological hallmark of PD [79]. However, the exact role of LBs in PD is still unclear. Recently, studies found that α-syn interacted with different isoforms of the NKA α subunit to mediate neuronal loss in PD [9,34]. A paper published in 2015 indicated that NKAα3 was a cell surface partner of α-syn aggregates (both oligomeric and fibrillar a-syn) [34]. When α-syn aggregates bound to and formed clusters at the plasma membrane of neurons, the freely diffusing NKAα3 was trapped within this cluster. α-syn aggregates interacted with NKAα3 and caused NKAα3 to become redistributed and form larger nanoclusters. This nanoclustering of NKAα3 created regions within the plasma membrane with reduced local densities of NKAα3. The α-syn/NKAα3 complex reduced the specific function of NKAα3: rapid Na^+^ extrusion from neurons and eventually resulted in neuronal loss by causing Ca^2+^ excitotoxicity (Figure 3) [34]. These data suggest that designing molecules derived from α-syn binding partners to interfere with α-syn binding to the neuronal plasma membrane, such as NKA-derived polypeptides, holds promise as a disease-modifying therapy for PD [80].

We recently reported that NKAα1 dysfunction in NKAα1^+/−^ mice exacerbated α-syn-induced tyrosine hydroxylase (TH) deficiency and behavioral problems in a preformed fibril (PFF)-induced mouse model of PD [9]. DR5-12D could reverse this phenomenon. It is an NKA-stabilizing monoclonal antibody that antagonizes the DR region of the NKAα1 subunit. We found that DR5-12D promotes α-syn degradation in neurons. The underlying mechanism for this effect included the activation of NKAα1-dependent autophagy via an AMPK/mTOR/ULK1 signaling pathway [9]. Cumulatively, this work demonstrated that NKA was neuroprotective for PD (Figure 3). Taken together, these data suggest that the neuronal α1 and α3 subunits of NKA are promising therapeutic targets, and drugs against NKA may represent a new therapeutic strategy for PD.

### 4.2. NKA and Mitochondrial Homeostasis

Mitochondria are significant organelles in cells. They are responsible for cellular respiration and produce ATP [81]. Reactive oxygen species (ROS) are the natural by-product of mitochondrial respiration. ROS damage DNA, lipids and proteins in the central nervous system [82]. Mitochondrial dysfunction and subsequent oxidative stress contributed to the loss of dopaminergic neurons in PD [83]. However, the mechanisms underlying these phenomena are still unclear. As an innate sensor for oxygen, NKA was highly sensitive to ROS and reacted promptly to energy deficiency in the brain [84]. The activity of NKA was reduced in a 1-Methyl-4-phenyl-1,2,3,6-tetrahydropyridine (MPTP)-induced mouse model of PD (about −40%) compared to the control group [85]. Consistent with these findings, the activity of NKA was also significantly decreased in 6-hydroxydopamine and rotenone-induced rodent models of PD (about −20~40%) [73,86]. In these rodent models, NKA dysfunction was caused by neurotoxin-induced mitochondrial dysfunction and then led to neuronal damage and death. Restoration of NKA activity in neurons by antioxidants, such as caffeine, hesperidin and quercetin, was suggested to relieve neuronal loss in SNpc and motor deficits in PD rodent models [73,85,86]. These studies illustrate the value and generality of NKA impairment and mitochondrial homeostasis in PD.

### 4.3. ATP1A3 Mutations and Rapid-Onset Dystonia–Parkinsonism (RDP)

Rapid-onset dystonia-parkinsonism (RDP) is an autosomal dominant disorder that is characterized by involuntary muscle contractions, abnormal posture and repetitive movements [87]. Although the symptoms are similar in RDP and PD, deep brain stimulation (DBS) and levodopa treatment have no effect on RDP patients [88,89]. *ATP1A3* (encoding the α3 subunit of NKA) mutations were first connected to RDP in 2004 [75]. Six novel missense mutations in the *ATP1A3* gene were identified and exhibited a strong correlation to RDP in seven families worldwide [75]. These mutations were located in the transmembrane (TM) region, TM domain-P domain, P domain-N domain and C-terminus of α subunit, which affected the affinity of ions to the NKA α3 subunit [75,90]. It is notable that more and more mutations were associated with RDP, each being related to different severity levels. Specifically, T163M was the most common and with the most severe outcome [91]. However, sporadic RDP may also not relate to any mutation in *ATP1A3* [92]. Comprehensively investigation of the function and molecular mechanisms of NKA α3 subunit would benefit the understanding of the pathogenesis of RDP.

## 5. The Role of NKA in Amyotrophic Lateral Sclerosis

Amyotrophic lateral sclerosis (ALS) is a neurodegenerative disorder characterized by progressive loss of motor neurons in the cerebral cortex. Most patients eventually succumb to respiratory failure [93]. Although genetic influences and environmental factors contribute to ALS pathogenesis, the etiology of ALS is still unknown [94]. It was found that the activity of NKA was decreased significantly in the motor neurons of Cu/Zn superoxide dismutase (SOD1)^G93A^ mice, which resulted in motor neurons degeneration [95]. Another report suggests that the activity of NKA was reduced remarkably in the spinal cord of SOD1^G93A^ mice, which contributed to ALS pathology [96]. These findings support the notion that NKA dysfunction plays a role in ALS [97].

Cu/Zn superoxide dismutase (SOD1) is encoded by *SOD1* gene. *SOD1* gene is the first gene that linked to ALS [98]. The mutation of *SOD1* alters ROS production and removal [98]. Transgenic mice carrying mutant SOD1^G93A^ are common model for ALS [99]. However, the specific effect of mutant SOD1 in ALS is still unknown. It was found that NKA was the ligand protein for mutant SOD1 [100]. In a shotgun proteomic analysis, three α subunits of NKA (α1, α2, α3) showed high probability scores and high peptide hits. These results indicate that NKA directly interacted with a mutant SOD1 [100]. Consistently, a misfolded state of SOD1 (misfSOD1) directly interacted onto NKAα3-specific 10 amino acid stretch to form a complex with NKAα3. The SOD1/NKA complex impaired the activity of NKA and further modified the expression level of glutamate receptor 2 (GluR2). Adeno associated virus (AAV)-mediated overexpression of NKAα3 delayed pathological alterations and prolonged survival of SOD1^G93A^ mice [101]. Briefly, these studies demonstrated that impairment in NKA activity was a determinant for selective vulnerability of motor neurons in ALS and provided valuable new avenues for potential therapeutic strategies for ALS [101].

The α1 and 3 subunits of NKA are expressed in neurons, the α2 subunit is astrocyte-specific [102]. Although, ALS is thought to selectively affect neurons, astrocytes are another target of ALS [102]. However, the molecular mechanism involved in astrocyte- induced neurodegeneration remains poorly understood. Evidence found that the expression level of NKAα2 was enhanced in astrocytes in ALS. Furthermore, it co-immunoprecipitated with α-adducin to form a complex [102]. The α-adducin is a cytoskeleton-associated protein, which regulates synaptic contacts [103]. This complex stimulated mitochondrial respiration to induce an inflammatory response. Inhibition of NKAα2 relieved motor neuronal loss and significantly enhanced mouse lifespan. Together, these findings suggest that astrocytic NKAα2 could induce neuronal loss [102]. However, the deficits of NKAα3 and NKAα1 in neurons aggravated the neuronal loss in ALS. These opposite results may stem from the cell-specific expression of the α subunit of NKA, a similar phenomenon also exists in AD. Understanding the mechanisms underlying isoform-specific patterns of the α subunit as the common feature of any neurodegenerative pathology, we can exploit the pharmacology of cell specific NKA to improve the outcome of NDDs.

## 6. The Role of NKA in Huntington’s Disease

Huntington’s disease (HD) is an autosomal-dominant neurodegenerative disease characterized by chorea, behavioral problems and cognitive dysfunction [104]. The expansion of CAG repeats in the *huntingtin* (*HTT)* gene is a major cause of HD. This gene encodes a polyglutamine (polyQ) stretch near the protein N-terminus, at which 36 repeats are a pathological threshold [105]. The precise mechanism of HD remains largely unclear. Evidence showed that HD was associated with a general membrane abnormality [106]. As a transmembrane protein, NKA dysfunction was also observed in HD patients [107]. One research team found that the activity of NKA was increased in HD patients compared with normal controls [106]. However, other studies did not find a higher NKA activity of erythrocytes from HD patients [108]. Studies also found that the NKA activity was reduced in lipid rafts (a kind of membrane structure) in the brain of HD patients [109]. The inconsistent activity of NKA observed in HD patients may stem from different cell types. Furthermore, the pathological mechanisms of HD patients are still not clear, and the etiology of HD vary between individuals, which may explain the difference in NKA activity.

**NKA and neuronal loss:** Extensive neuronal cell loss is a hallmark of HD. Disturbing cell death pathways slows the development of HD. However, it is difficult to rescue neuronal loss in HD [110]. It is interesting to observe that NKA dysfunction promotes neuronal loss via different mechanisms. Firstly, N-methyl-D-aspartate receptors (NMDARs) play an essential role in excitatory neurotransmission in the brain [111]. Overactivation of NMDAR resulted in excitotoxicity, which was associated with HD [112]. NMDAR and other ionotropic neuronal receptors need NKA to maintain a resting membrane potential between −70 and −80 mV. However, impaired mitochondria in HD produced less ATP to synthesize NKA, which led to cell depolarization [113]. Under normal resting potential, Mg^2+^ ions blocked the pore of NMDAR. However, when the membrane was depolarized, the Mg^2+^ left NMDAR and allowed free entry of Ca^2+^, which eventually led to neuronal loss [114]. Furthermore, a strong correlation between NKA deficiency and oxidative stress was reported in HD. Studies showed that the activity of δ-ALA-D was significantly decreased during HD-like symptoms induced by 3-nitropropionic acid (3-NP) in rats [115]. The depletion of δ-ALA-D activity resulted in the accumulation of δ-ALA, an inhibitor of NKA. Since NKA maintained membrane potential, its inhibition induced neuronal loss in these rats [112]. These findings support the notion that NKA deficiency contributes to neuronal loss in HD. In line with this, some antioxidants, such as thymoquinone, exerted their neuroprotective effect by restoring the activity of NKA in HD [116].

## 7. The Role of NKA in Multiple Sclerosis

Multiple sclerosis (MS) is a complex inflammatory and demyelinating disorder in the central nervous system [117]. In MS, the immune system attacks the myelin that covers neurons and disrupts the axon membrane. In the demyelinated region of neuronal axons, conduction is delayed or possibly blocked entirely. Failure of conduction in chronically demyelinated axons induces functional deficits in MS [118]. Body functions supervised by these affected neurons are gradually deteriorating. However, the recovery of conduction in the absence of remyelination has been observed by some research groups, and the mechanism underlying this phenomenon is not fully understood.

In active plaques (relatively recent plaques), the activity of NKA was significantly decreased, whereas in inactive plaques (old, chronic plaques), the activity of NKA was increased compared to normal [119]. The loss and subsequent restoration of NKA activity may stem from the altered composition of these plaques, where new axons and astrocytes (either or both may be richer in ATPase) made up the bulk of inactive plaques [119]. These findings in MS tissues offered an explanation for recovery of the conduction during remission. Meanwhile, these findings also indicate that NKA dysfunction was involved in MS. Furthermore, NKA dysfunction led to intracellular Na^+^ accumulation. Increased intracellular Na^+^ concentration triggered a cascade of events that ultimately resulted in neuroaxonal loss in MS [120]. Sodium magnetic resonance imaging (^23^Na-MRI) is an emerging imaging technique for quantifying brain tissue Na^+^ concentration in vivo, which reflects neuroaxonal integrity and metabolic function. Hence, this technique should be considered as a measure for diagnosing MS [121].

Although NKA activity was reduced in neurons in MS [122], there were several studies suggesting that inhibition of NKA affected this disease in an opposite fashion [123,124]. Firstly, activation and accumulation of autoreactive CD4^+^ T cells is a hallmark of MS [125]. However, how autoreactive CD4^+^ T cells recognize their target antigen is incompletely understood. A paper published in 2019 indicate that CD4^+^ T cells required ATG-dependent phagocytosis in dendritic cells (DCs) to recognize their target antigen. The NKA inhibitor neriifolin ameliorated myelin presentation to CD4^+^ T cells by inhibiting this pathway. Thus, pharmacological inhibition of NKA delayed the onset and reduced the clinical severity of MS [123]. Secondly, myelin basic protein (MBP) is a major component of myelin. The MBP deficiency leads to severe hypomyelination in MS. It was found that ouabain stimulated MBP synthesis by blocking NKAα2-dependent Na^+^/Ca^2+^ exchanger-mediated spontaneous Ca^2+^ transients in oligodendrocytes. In line with this, the knockdown of NKAα2 with small interfering (si)RNA (α2-siRNA) significantly promoted MBP synthesis in active axons [124]. Hence, these data indicate that the role of NKA in a variety of cell types in the brain may be distinct and that NKA should be considered as a potential detector of neuronal activity in MS.

## 8. Conclusions and Future Directions

As an ion pump, the function of NKA in NDDs is well recognized by the scientific research community. It maintains membrane potential and thus maintains neuronal excitability, but it also regulates many essential cellular functions to modulate neuronal loss in NDDs. However, there are some barriers limiting its widespread application. First of all, three isoforms of the NKA α subunit are expressed in the brain. As mentioned above, the dysfunction of these isoforms plays an important role in most NDDs. In particular, α1/α3 subunit deficiency accelerates neuronal loss by regulating a plethora of signaling cascades and reduces pump function. In contrast, the overactivated α2 subunit facilitates neuronal loss and the progress of ALS and AD. This seemingly paradoxical phenomenon shows that precision therapies targeted for different isoforms of the NKA α subunit in NDDs are necessary. Secondly, NDDs are no longer cell-autonomous diseases only affecting neurons. We should consider them as a disruption of the CNS environment. Different cell–cell interactions and cell signaling events in the CNS environment play important roles in the development of NDDs. Thus, a holistic understanding of these isoforms in different cells may be harnessed to improve therapeutics for neurodegeneration. Thirdly, current research mainly focuses on the α subunit of NKA. There are too few clinical/preclinical experimental studies exploring the role of the β subunit in NDDs. Although it does not form the transport pore, the β subunit influences the stability and function of the α subunit in the plasma membrane. The β subunit may play an important role in the regulation of the interaction between pathogenic proteins and the α subunit of NKA. Hence, there is a great need for adequate and in-depth research on the β subunit in NDDs.

Collectively, this review summarized and discussed the therapy roles and the underlying mechanisms of NKA in NDDs. For instance, our group found that the NKAα1-dependent autophagic flux was inhibited in a PFF-induced PD mouse model. We generated a monoclonal antibody against the DR region of the NKA α subunit, DR5-12D. DR5-12D restores the autophagy flux to alleviate the learning and memory impairment through inhibition of the formation of the NKAα1/AMPKα/α-syn complex. However, more novel insights into neuronal loss are urgently required because of the increased incidence of NDDs. With more and more exciting findings regarding the mechanistic network of NKA in NDDs, we expect the development of multiple innovative NKA therapeutics for NDDs in the near future.

## Data Availability

The data presented in this study are available in the article.
